# Hybrid transformer convolutional neural network-based radiomics models for osteoporosis screening in routine CT

**DOI:** 10.1186/s12880-024-01240-5

**Published:** 2024-03-14

**Authors:** Jiachen Liu, Huan Wang, Xiuqi Shan, Lei Zhang, Shaoqian Cui, Zelin Shi, Yunpeng Liu, Yingdi Zhang, Lanbo Wang

**Affiliations:** 1 Department of Orthopedics, Shengjing Hospital of China Medical University, 110004 Shenyang, People’s Republic of China; 2 Department of Radiology, Shengjing Hospital of China Medical University, 110004 Shenyang, People’s Republic of China; 3 Shenyang Institute of Automation, Chinese Academy of Sciences, 110016 Shenyang, People’s Republic of China

**Keywords:** Radiomics, Vertebrae, Convolutional neural network, Osteoporosis, Transformer

## Abstract

**Objective:**

Early diagnosis of osteoporosis is crucial to prevent osteoporotic vertebral fracture and complications of spine surgery. We aimed to conduct a hybrid transformer convolutional neural network (HTCNN)-based radiomics model for osteoporosis screening in routine CT.

**Methods:**

To investigate the HTCNN algorithm for vertebrae and trabecular segmentation, 92 training subjects and 45 test subjects were employed. Furthermore, we included 283 vertebral bodies and randomly divided them into the training cohort (*n* = 204) and test cohort (*n* = 79) for radiomics analysis. Area receiver operating characteristic curves (AUCs) and decision curve analysis (DCA) were applied to compare the performance and clinical value between radiomics models and Hounsfield Unit (HU) values to detect dual-energy X-ray absorptiometry (DXA) based osteoporosis.

**Results:**

HTCNN algorithm revealed high precision for the segmentation of the vertebral body and trabecular compartment. In test sets, the mean dice scores reach 0.968 and 0.961. 12 features from the trabecular compartment and 15 features from the entire vertebral body were used to calculate the radiomics score (rad score). Compared with HU values and trabecular rad-score, the vertebrae rad-score suggested the best efficacy for osteoporosis and non-osteoporosis discrimination (training group: AUC = 0.95, 95%CI 0.91–0.99; test group: AUC = 0.97, 95%CI 0.93–1.00) and the differences were significant in test group according to the DeLong test (*p* < 0.05).

**Conclusions:**

This retrospective study demonstrated the superiority of the HTCNN-based vertebrae radiomics model for osteoporosis discrimination in routine CT.

**Supplementary Information:**

The online version contains supplementary material available at 10.1186/s12880-024-01240-5.

## Introduction

Osteoporosis is a common metabolic bone disease characterized by a progressive bone mass and strength reduction, which predisposes to an increased risk of fractures [1]. Over 32.0% of the population older than 65 years old suffer from the disease worldwide. Moreover, vertebra osteoporosis has been treated as a key factor for internal fixation system loosening and failure of spine fusion [2, 3]. Thus, better knowledge of bone mineral density (BMD) in vertebrae is essential to identify high-risk populations of fractures and reduce surgical complications.

Due to extra radiation exposure and medical costs, dual-energy X-ray absorptiometry (DXA) examinations were not routinely performed in patients with suspected osteoporosis before spine surgery in a survey [4]. Previous studies have suggested that the average Hounsfield unit (HU) values in vertebrae are significantly correlated with the T score obtained from DXA [5], which also can be utilized to detect osteoporosis and predict pedicle screw loosening [6]. However, HU values can vary depending on the CT protocol, and the measurement of HU in routine CT is subject to user-dependent definitions of regions of interest (ROIs) that may not capture all radiologic information from vertebral bodies [7].

Radiomics, quantitative features capturing the non-vision image information, have been applied for disease diagnosis and prediction [8]. Accumulating evidence has confirmed the potential classification performance for bone diseases [9, 10]. Recent studies suggested the excellent performance of radiomics models for osteoporosis screening and fracture prediction [11, 12].

With the rapid development of deep learning algorithms, medical image recognition has witnessed a surge of interest in the application of convolutional neural networks [13]. In contrast, the transformer network, which features a distinct architecture from convolution, is emerging as a promising technology for analyzing medical images, as it can enable CNN to capture long-distance relationships from images [14, 15]. The transformer network learns the relationships among features, leading to a more versatile model that is not entirely reliant on the training data. Moreover, transformers have exhibited high accuracy when employed in various medical tasks [16, 17]. Thus, we developed a hybrid architecture that combines deep learning and transformer algorithms for automated vertebral body and trabecular compartment segmentation.

In this study, we proposed a transformer-enhanced deep learning framework to automatically acquire features from the entire lumbar vertebral body and the cancellous compartment to develop radiomics models for osteoporosis screening in routine CT.

## Materials and methods

### Study patient population

**Fig. 1:**
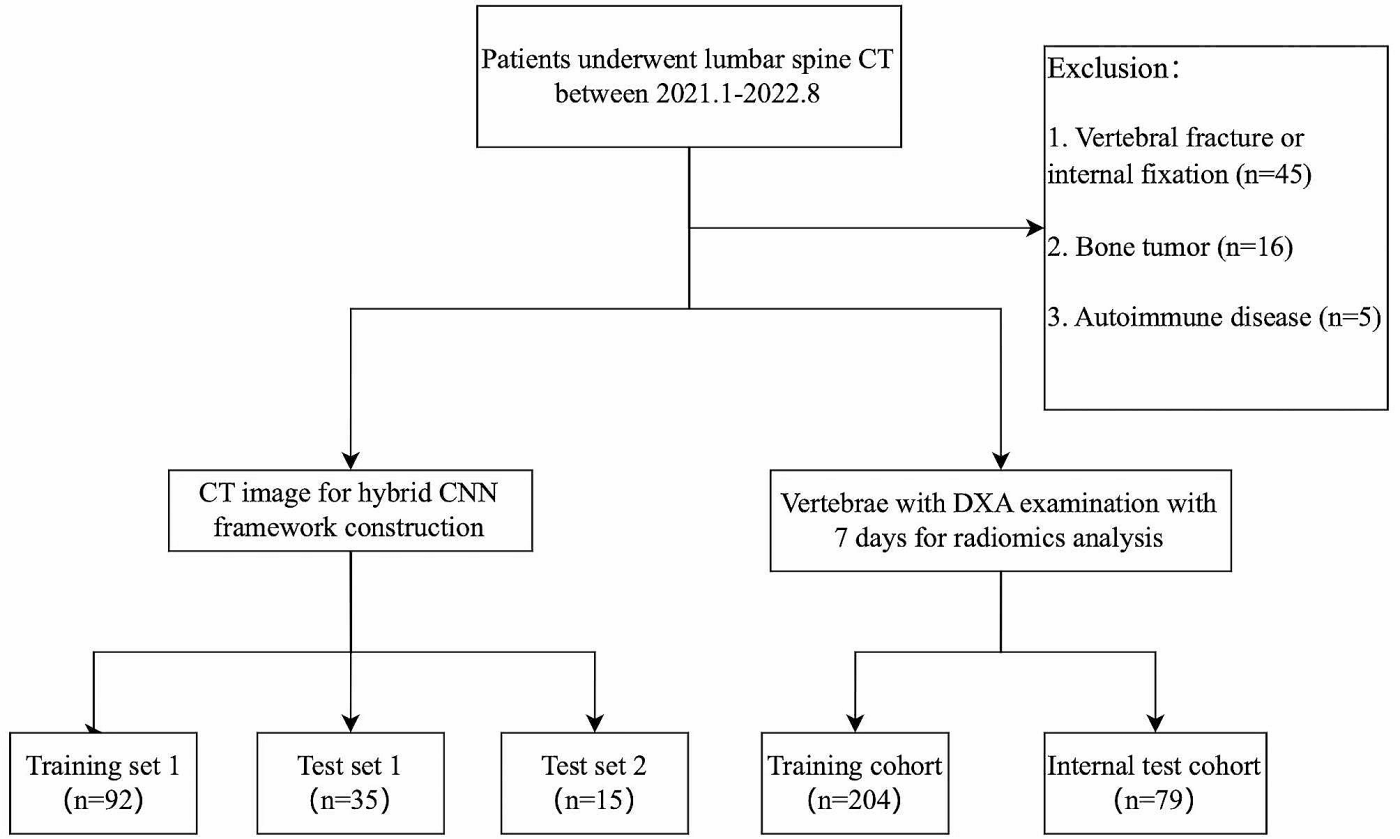
Flowchart shows the process of patient recruitment and exclusion

Between January 2021 and August 2022, we retrospectively searched consecutive patients undergoing multi-detector CT (MDCT) scanners from the institutional image database. This retrospective study was approved by the Ethics Committee of Shengjing Hospital of China Medical University, and the need for informed consent was waived. The inclusion criteria were (1) patients with ages older than 18; (2) available lumbar spine CT data. The exclusion criteria were as follows: (1) vertebral fracture and internal fixation history; (2) bone tumor; (3) vertebrae with severe degenerative changes; (4) hematological disorders and autoimmune diseases. Among them, patients with DEXA results and lumbar spine CT images within seven days were selected for radiomics analysis. The selection pipeline is presented in Fig. 1. Following the WHO criteria [18], lumbar vertebrae (L1-L4) were classified into DXA-based categories: non-osteoporosis with a T-score > − 2.5; osteoporosis with a T-score ≤ − 2.5. Vertebrae were divided into train and internal validation cohorts using a stratified random sampling algorithm at a 7:3 ratio.

### CT image acquisition

The images were obtained from six different MDCT scanners in our hospital with a peak tube voltage of 120 kV (Ingenuity Core 128 and iCT 256, Philips Systems; Somatom Definition and Sensation 64, Siemens Systems). Images were retrieved from the Picture Archiving and Communication Systems (PACS) in the DICOM format and reconstructed at a slice thickness of 1 to 1.5 mm. The DEXA was performed in the lumbar spine and the T-score of each vertebra (L1-4) was obtained from the report.

### Image annotation and hybrid transformer convolutional neural network training

The entire vertebral body and cancellous compartment of the vertebral body were segmented manually slice by slice in ITK-SNAP software (version 3.6.0, www.itksnap.org). Two residents, who have been specifically instructed and trained, conducted the initial manual segmentation. To investigate the intraclass variability, another resident (board-certified radiologist) performed duplicated annotation in randomly selected images, who was blind to the previous annotation results. We utilized the Inter Correlation Coefficient between two experienced residents to assess the repeatability of segmentation. We proposed a novel deep learning algorithm, ST-Unet, an architecture with efficient integration of Swin-Transformer and 3D U-Net algorithm. Details on ST-Unet training are available in Supplementary Materials. The overall segmentation performance is quantitatively evaluated by two metrics, the dice similarity coefficient (DSC) and the average surface distance (ASD).

### HU measurement and radiomics features extraction

**Fig. 2:**
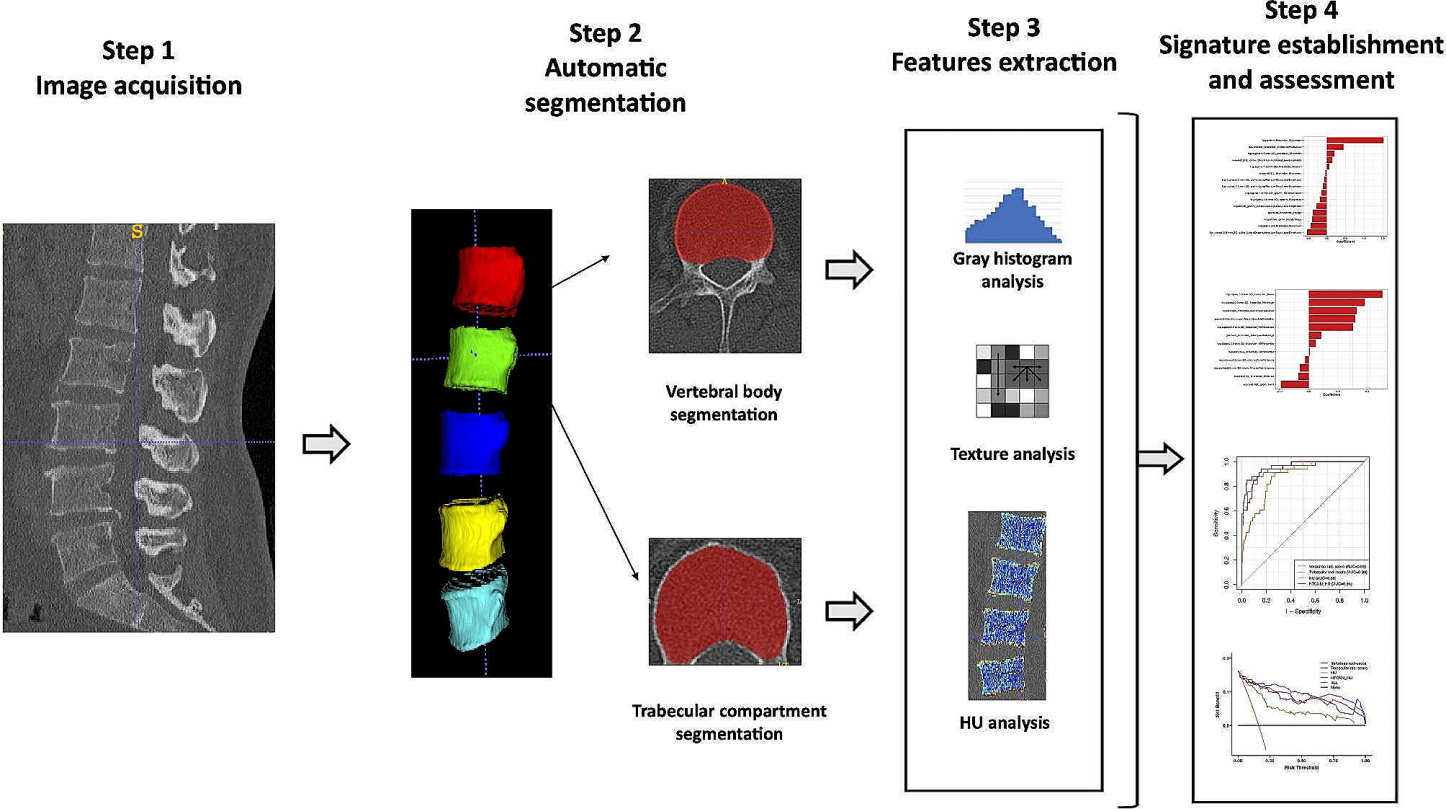
Flow diagram for the construction of radiomics model based on the automatic segmentation of vertebral body and trabecular compartment

HU values were manually measured from the region of anterior cancellous compartments of vertebral bodies (L1-4) in PACS [19]. Additionally, we automatically extracted the average HU (HTCNN_HU) from the segmentation masks of the cancellous region. Radiomics features were extracted from two masks in L1-4 using the PyRadiomics package [20] in Python (version 3.7) after z-score normalization and resampling of images, consisting of six classes: first-order statistics, gray-level co-occurrence matrix (GLCM), gray-level run length matrix (GLRLM), neighboring gray-tone difference matrix (NGTDM), gray-level size zone matrix (GLSZM) and gray-level dependence matrix (GLDM). In addition, seven transform filters including wavelet and Log were applied for feature extraction. The muti-step workflow is present in Fig. 2.

### Radiomics signature establishment and evaluation

First, we normalized the radiomics features and removed zero variation features by the“preProcess” package in R. Second, highly correlated feature clusters with correlation coefficients > 0.9 were collapsed into the representative feature. Third, the radiomics signature for osteoporosis discrimination was established using the random forests (RF) and least absolute shrinkage and selection operator (LASSO) regression algorithm with 10-fold cross-validation using “RandomForest” and “glmnet” packages. Fourth, we calculated the radiomics score (rad-score) according to a linear combination of the selected features and their respective LASSO coefficients. ROC curves were applied to assess the classification performance of radiomics signatures and HU values in the training and test sets. The area under the ROC (AUC) was calculated using “pROC” package. Finally, we estimated the clinical value through the decision curve analysis (DCA).

### Statistical analysis

All statistical analyses were performed using R (version 3.4.2; http://www.Rproject.org). The chi-square test (categorical variables) and Student t test (continuous variables) were conducted to compare the clinical characteristics. *P* < 0.05 suggested a statistically significant difference. The DeLong test was applied to compare the difference in AUC.

## Results

### Clinical characteristics

The automated segmentation framework was constructed using a training cohort including CT data from 92 patients. Test cohorts were from two different campuses of our hospital consisting of 35 and 15 patients respectively. A total of 283 vertebrae were eligible for radiomics analysis in this research. We randomly divided the included subjects into a training cohort (*N* = 204) and a test cohort (*N* = 79). The clinical characteristics of patients for radiomics analysis are listed in Table 1.

**Table 1:** Clinical characteristics in the training and test sets for radiomics analysis

	Training cohort (*n* = 204)			Test cohort (*n* = 79)			*P*
	Osteoporosis	Non-osteoporosis	*P*	Osteoporosis	Non-osteoporosis	*P*	
Sex			0.001^*^			0.827	0.499
Male	3	68		6	28		
Female	30	103		10	35		
Age	63.64.12 ± 7.47	59.13 ± 9.76	0.012^*^	63.56 ± 9.83	61.03 ± 9.95	0.365	0.063
HU	72.02 ± 33.25	137.10 ± 45.94	*P* < 0.001^*^	83.14 ± 41.89	120.10 ± 40.20	0.002^*^	0.030^*^
HTCNN_ HU	108.10 ± 29.84	182.90.1 ± 42.24	*P* < 0.001^*^	122.90 ± 34.97	167.00 ± 36.48	*P* < 0.001^*^	0.039^*^

Data are mean ± standard deviation.

HTCNN, hybrid transformer deep convolutional neural network; HU, Hounsfield unit.

*P value < 0.05

### Performance assessment of the segmentation framework

The interrater variability is reported in Table 2 and Supplementary Data 1. The performance of HTCNN is summarized in Table 2. The results suggested high agreement between the automatic framework and manual segmentation with mean DSCs of 0.968, ASD of 0.481 for vertebral body segmentation, and mean DSCs of 0.961, ASD of 0.494 for trabecular compartment segmentation in test cohorts.

**Table 2:** Performance of automatic segmentation

Vertebral body	Interrater(*n* = 5)	Test1 (*n* = 35)		Test2 (*n* = 15)	
	DSCs	DSCs	ASD	DSCs	ASD
L1	0.965 ± 0.015	0.964 ± 0.012	0.605 ± 0.521	0.965 ± 0.05	0.452 ± 0.069
L2	0.969 ± 0.009	0.956 ± 0.048	0.772 ± 1.953	0.967 ± 0.05	0.432 ± 0.086
L3	0.967 ± 0.016	0.969 ± 0.008	0.367 ± 0.165	0.965 ± 0.010	0.467 ± 0.142
L4	0.943 ± 0.060	0.973 ± 0.010	0.336 ± 0.139	0.976 ± 0.009	0.422 ± 0.196
Trabecular compartment of the vertebral body					
L1	0.962 ± 0.010	0.966 ± 0.022	0.431 ± 0.443	0.960 ± 0.012	0.539 ± 0.552
L2	0.968 ± 0.011	0.966 ± 0.024	0.445 ± 0.486	0.959 ± 0.017	0.706 ± 0.911
L3	0.968 ± 0.008	0.963 ± 0.036	0.387 ± 0.266	0.959 ± 0.012	0.511 ± 0.604
L4	0.966 ± 0.010	0.962 ± 0.049	0.430 ± 0.385	0.955 ± 0.032	0.507 ± 0.496

Mean dice similarity coefficient and average surface distance (± standard deviation) are used to evaluate the performance of the automatic segmentation framework

DSCs, dice similarity coefficient; ASD, average surface distance

### Features selection and radiomics signature establishment

**Fig. 3:**
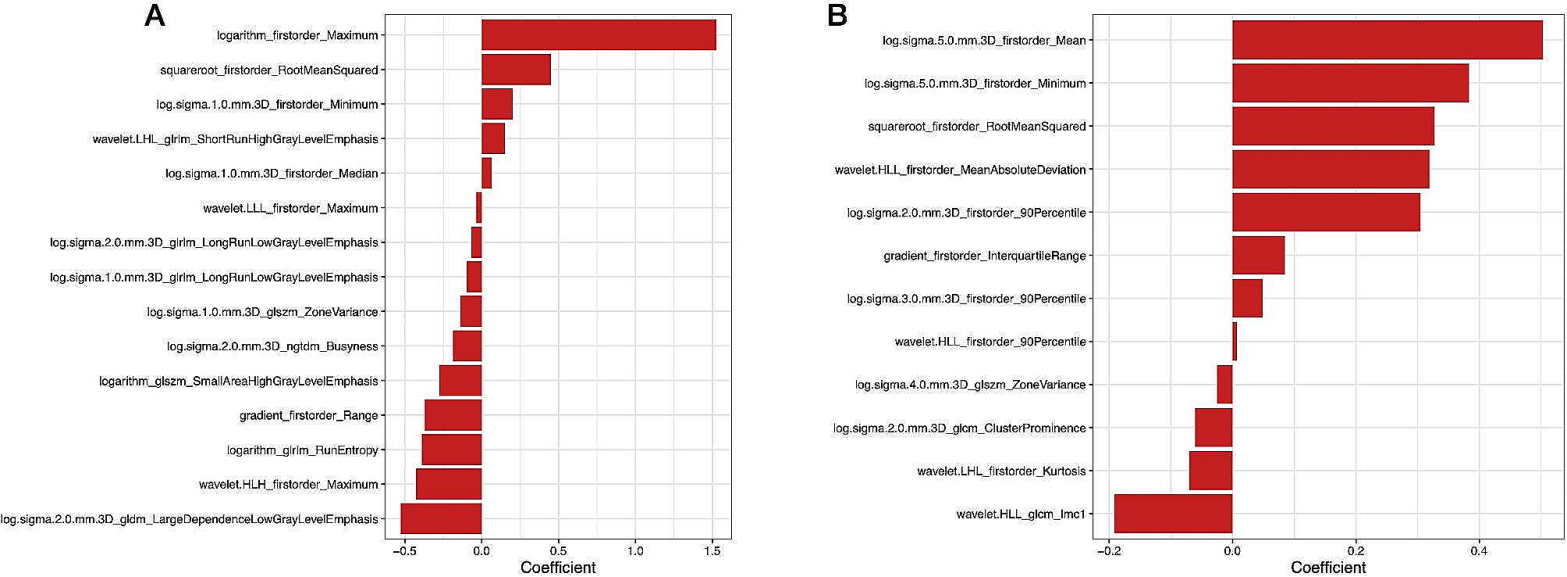
Histogram illustrates the optimal feature signature and their weights for the vertebrae radiomics model (**A**) and trabecular radiomics model (**B**)

A total of 1767 features were extracted from two ROIs respectively. First, excluding zero variance features and highly correlated features, 520 features from the trabecular region and 473 features from the entire vertebral body region were retained for RF selection. Second, the 30 features with the highest importance according to Gini coefficients were screened for signature construction (Supplementary Materials). Third, we calculated the vertebrae and trabecular rad-score according to the optimal radiomics signature selected by LASSO regression with tenfold cross-validation (Fig. 3) (Supplementary Materials).

### Assessment and validation of the radiomics model

**Fig. 4:**
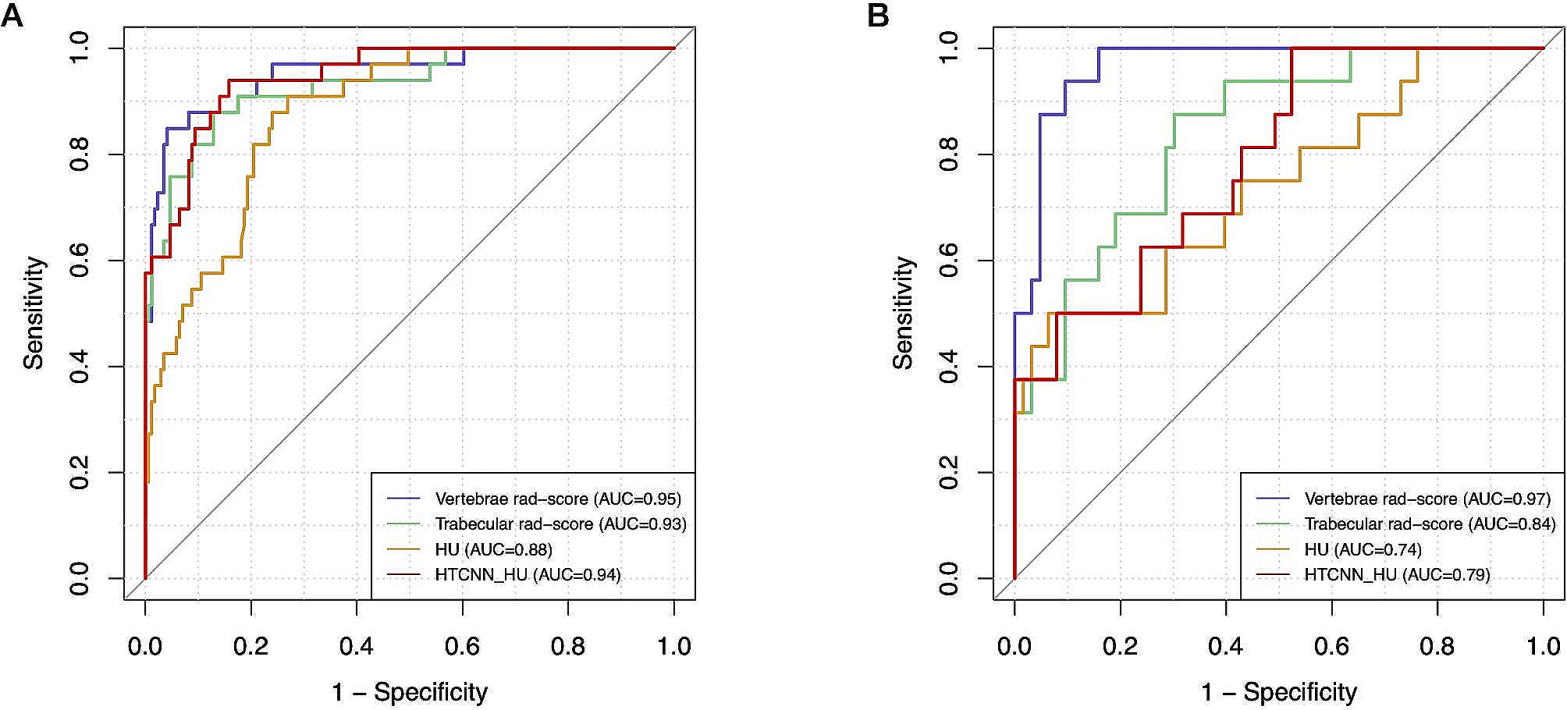
Comparison of the discrimination ability between vertebrae radiomics score, trabecular radiomics score, HU, and HTCNN_HU in training (**A**) and validation cohort (**B**)

**Fig. 5:**
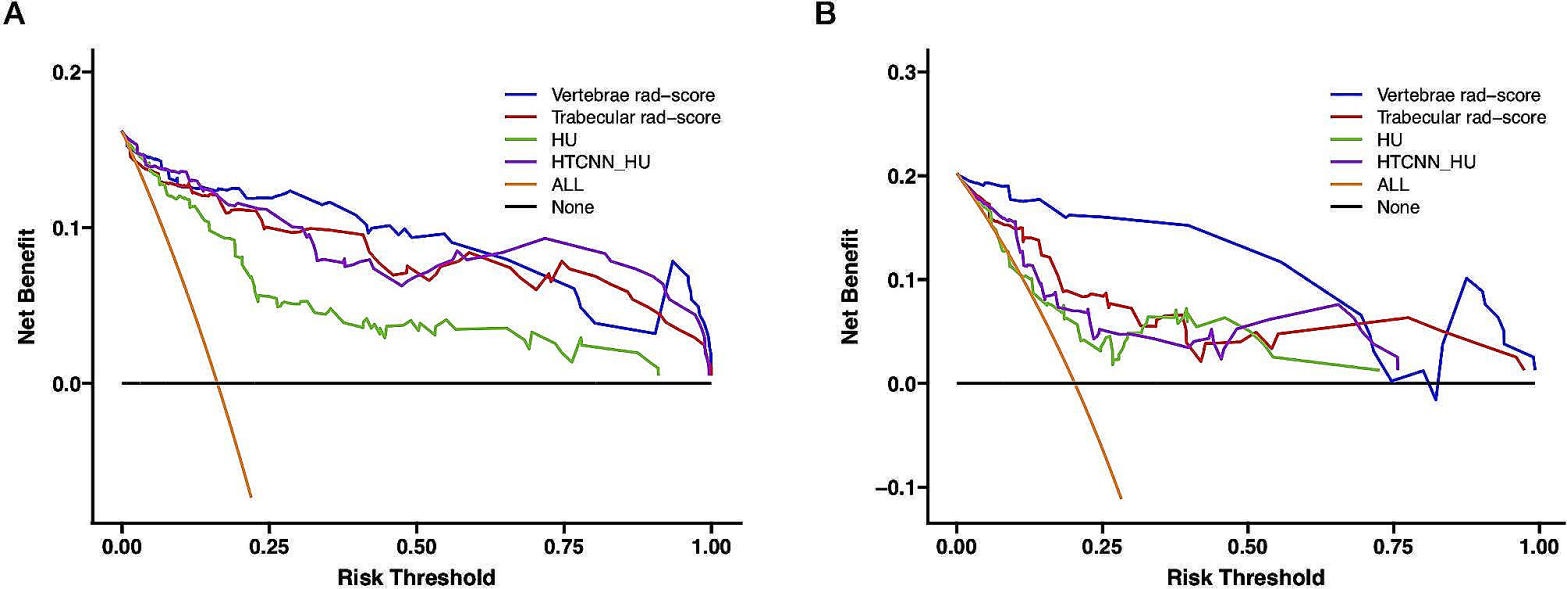
Comparison of the clinical applicability between vertebrae radiomics score, trabecular radiomics score, HU, and HTCNN_HU in training (**A**) and validation cohort (**B**)

ROC curves of rad-score and HU for osteoporosis prediction were presented in Fig. 4. In terms of the discriminatory power for osteoporosis, vertebrae rad-score yielded the best performance (training AUC = 0.95, 95%CI 0.91–0.99, test AUC = 0.97, 95%CI 0.93–1.00), which was significantly superior to trabecular rad-score, HTCNN_HU, and HU in the test cohort (*p* < 0.01). Additionally, the trabecular rad-score showed considerably higher accuracy than HTCNN_ HU and HU in the test set (AUC = 0.84 vs. 0.79, 0.74, respectively, *p* < 0.01 each, Table 3). The clinical utility of the four predictors was evaluated using decision curve analysis (DCA), which revealed that the vertebrae rad-score conferred the highest net benefit (Fig. 5).

**Table 3:** The discriminative power of osteoporosis for four predictors

Predictors	Group	AUC (95% CI)	Accuracy	Sensitivity	Specificity	PPV	NPV
HU	Train	0.88 (0.82–0.93)	0.76	0.73	0.91	0.98	0.40
	Test	0.74 (0.59–0.89)	0.85	0.94	0.50	0.89	0.67
HTCNN_ HU	Train	0.94 (0.91–0.98)	0.86	0.84	0.94	0.99	0.53
	Test	0.79 (0.67–0.91)	0.58	0.48	1.00	1.00	0.33
Trabecular rad-score	Train	0.93 (0.88–0.98)	0.87	0.88	0.87	0.57	0.97
	Test	0.84 (0.74–0.94)	0.73	0.88	0.70	0.42	0.96
Vertebrae rad-score	Train	0.95 (0.91–0.99)	0.94	0.85	0.96	0.80	0.97
	Test	0.97 (0.93-1.00)	0.91	0.94	0.91	0.71	0.98

PPV, positive predictive value; NPV, negative predictive value; HTCNN, hybrid transformer deep convolutional neural network; HU, Hounsfield unit

## Discussion

In this study, we developed a hybrid deep learning algorithm for automated 3D segmentation of the entire lumbar vertebral body and trabecular compartment in routine CT, which yielded high accuracy in different test sets. Compared with HU values and trabecular rad-score, the vertebrae rad-score revealed the highest diagnostic performance. The HTCNN-based vertebrae radiomics model can be served as a supportive tool for automated osteoporosis discrimination.

HU measurement is a common method for opportunistic osteoporosis screening which can be performed directly in the PACS [21]. Comparing with noncalibrated HU values is crucial to explore a more precise way for opportunistic osteoporosis screening. However, the manual measurement of HU values might not embrace the whole trabecular region. To address this limitation, we also performed HTCNN_HU measurement. Nevertheless, HTCNN_HU values are device-dependent, and the cut-off values for osteoporosis screening are inapplicable for other CT scanners with different imaging protocols [22]. Thus, we established radiomics signatures employing CT images from various scanning environments to maintain robustness.

ROI segmentation is a necessary step for radiomics analysis. Jiang et al. constructed a radiomics model to detect osteoporosis based on lumbar vertebral bodies [11]. However, the selected cohorts stem from the same CT scanner meanwhile the semi-automated labeling method is not accurate for automated segmentation of intact vertebral body. Xie et al. combined radiomic features from the cancellous compartment of L3 vertebrae in quantitative computed tomography (QCT) images and clinical information to discriminate osteoporosis and osteopenia [23]. Yet, only L3 vertebrae were considered in radiomics analysis and the labeling of ROI was performed by manual segmentation, which was time-consuming in the case of processing large image data. Compared with manual or semi-automatic segmentation, automated segmentation is thought to be more efficient and reproducible for radiomics analysis [24].

CNN and transformer have been increasingly applied to automated vertebrae recognization [25, 26]. Fang et al. established a vertebral body segmentation model for osteoporosis prediction using the 2D U-net algorithm, with a mean DSC of 0.85 [27]. Our hybrid architecture suggested superior accuracy in vertebral body segmentation compared to conventional CNN. Moreover, automated osteoporosis screening with deep learning has attracted attention, given that it obviates extra examinations by DXA or QCT. Yasaka et al. indicated significant correlations between CNN-based area BMD and DXA scores in both internal and external validation groups [28]. Additionally, volume BMD automatically extracted from routine CT showed a strong correlation with QCT-derived volume BMD [29]. Wang et al. developed a deep-learning model with a transformer encoder to estimate lumbar BMD from chest X-rays, which achieves high accuracy in osteoporosis classification [30]. Our findings confirm the high performance of the HTCNN-based radiomics model for osteoporosis screening in routine CT. This fully automatic pipeline might be implemented into a software program applied in the clinical scenario.

Several limitations should be acknowledged. First, in this single-center retrospective study, we only validated the performance of HTCNN-based radiomics models in the internal cohort, though image data was derived from six routine CT scanners with different protocols. Thus, multicenter studies with prospective designs are required to validate the generalizability of radiomics models. Second, similar to the previous CNN framework for vertebrae segmentation, vertebrae with severe fractures, degenerative changes, and implantation materials were excluded. Third, normalization protocols are required to maintain the robustness of radiomics, as the technical acquisition and parameter settings of CT images from different institutions contribute to deviations in radiomic features. Supplementary approaches for feature extraction or image conversion are feasible options [31, 32].

In conclusion, for automated osteoporosis screening in routine CT, we constructed and validated a HTCNN-based vertebrae radiomics model with high efficacy, which can promote clinical decisions and supplement the current screening system.

### Electronic supplementary material

Below is the link to the electronic supplementary material.

Supplementary Material 1

Supplementary Material 2

## Data Availability

All data generated or analyzed during this study are included in this published article.
