# Supplementary material for: Hybrid transformer convolutional neural network-based radiomics models for osteoporosis screening in routine CT
Source: BMC Med Imaging. 2024 Mar 14;24:62. doi: 10.1186/s12880-024-01240-5 (PMC10938662; doi:10.1186/s12880-024-01240-5)
Supplement: Supplementary file 1 — Supplementary Material 1 [file 12880_2024_1240_MOESM1_ESM.docx]

**Supplementary Material**

**The detail of hybrid CNN architecture**

The network is built on the standard encoder-decoder architecture. Specifically, for the encoder, different from pure 3D-CNN [1] or Transformer approaches [2] [3], a hybrid structure consisting of CNN and Swin-Transformer is employed to hierarchically extract the feature. And for the decoder, we apply the form of UNETR [2], which consists of multiple residual convolutional layers and transposed-convolutional layers for up-sampling.

**Loss function**

We adopt the summation of Dice loss and cross-entropy loss with equal weight as loss function.

**Implementation details**

The model is implemented based on Pytorch and MONAI and trained on NVIDIA 3090 GPU. The input image size is resized to 224×224×64 for training with the batch size equal to 1. We use SGD optimizer with Nesterov momentum. All networks are trained for 1000 epochs with one epoch including 250 mini-batches. We use an initial learning rate of 0.01 and a polynomial learning rate policy.

**Supplementary Figure**

Supplementary Figure 1. Radiomics feature selection from vertebral body region using minimum random forest (RF) and least absolute shrinkage and selection operator (LASSO). (A) RF was performed to eliminate the redundant and irrelevant features. (B) LASSO method was used to confirm the optimal adjustment parameter λ, and the vertical line was drawn according to the value selected by 10-fold cross-validation. (C) LASSO coefficient profiles of the radiomics features. A coefficient profile plot was produced against the Log (λ) sequence.

**
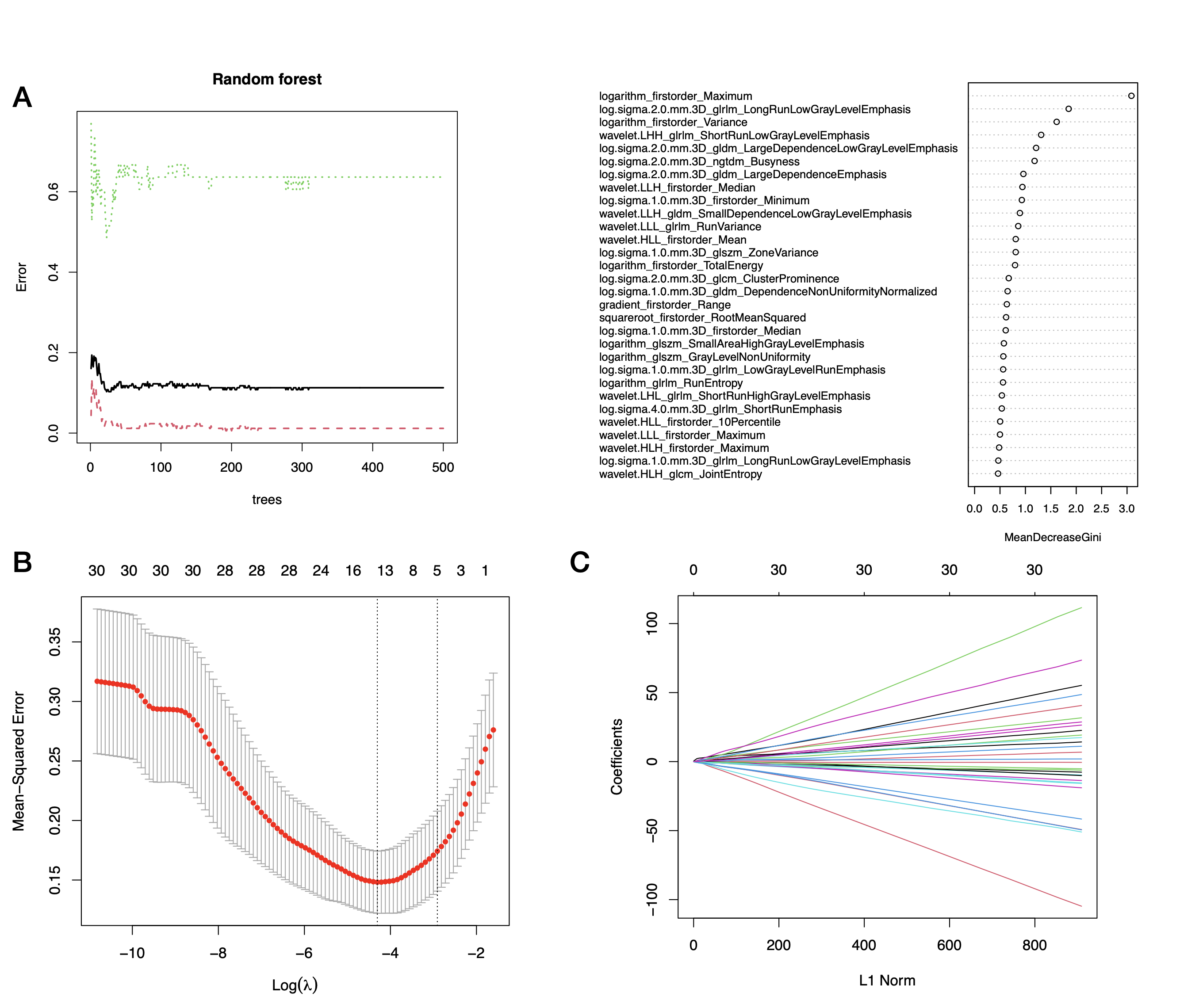
**

Supplementary Figure 2. Radiomics feature selection from the cancellous bone region using minimum random forest (RF) and least absolute shrinkage and selection operator (LASSO). (A) RF was performed to eliminate the redundant and irrelevant features. (B) LASSO method was used to confirm the optimal adjustment parameter λ, and the vertical line was drawn according to the value selected by 10-fold cross-validation. (C) LASSO coefficient profiles of the radiomics features. A coefficient profile plot was produced against the Log (λ) sequence.

**
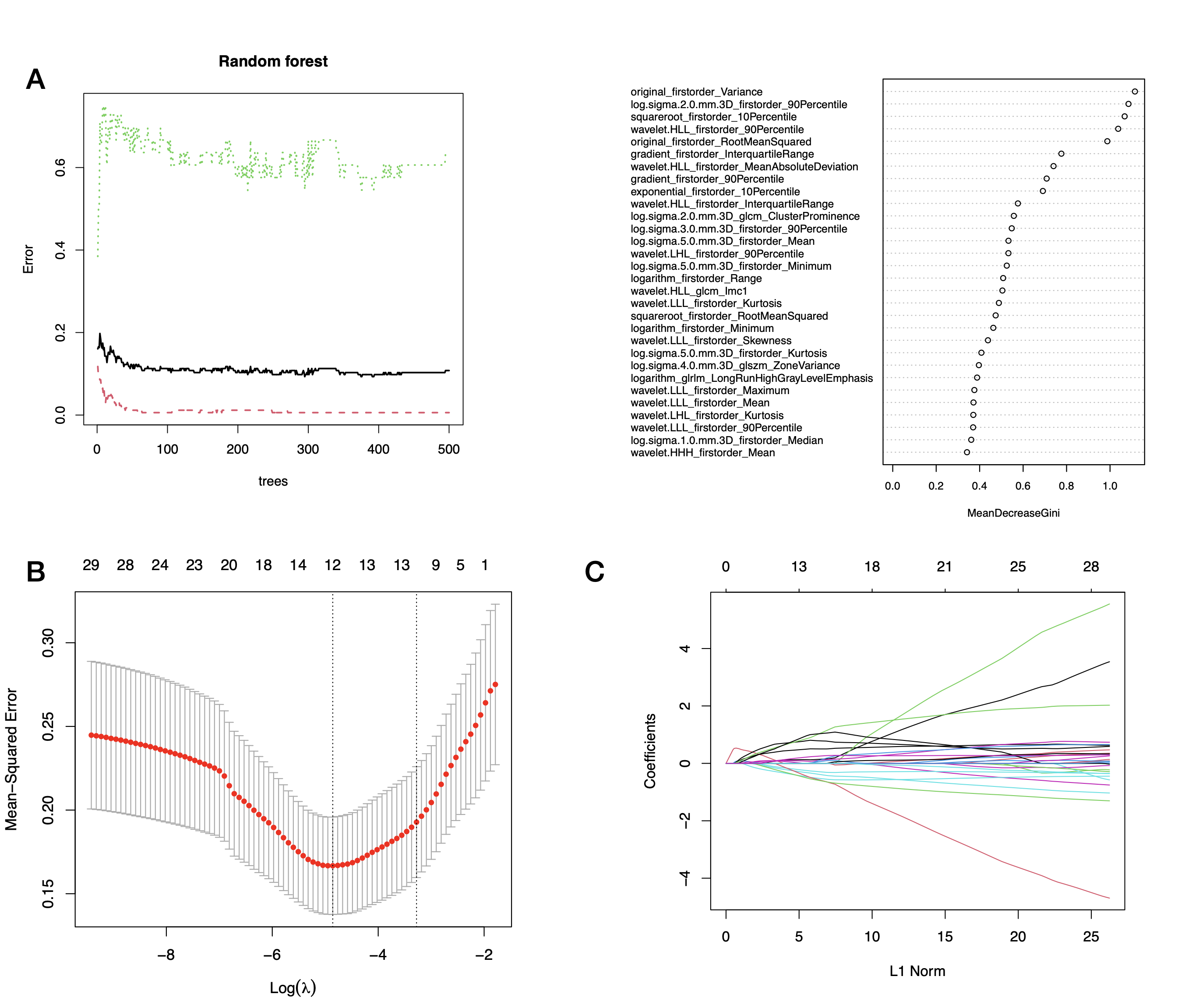
**

**Supplementary Table**

**Table S1** Comparison to state-of-the-art methods

| Method | DSC$\uparrow$(%) | ASD$\downarrow$ |
| --- | --- | --- |
| 3DUNet | 86.15 | 3.488 |
| VNet | 85.52 | 1.396 |
| AttentionUNet | 87.32 | 1.238 |
| UNETR | 87.08 | 1.531 |
| Swin-UNETR | 88.00 | 1.277 |
| Ours | **96.5** | **0.49** |

| **Table S2** Selected Radiomics Features and Their Weights for Vertebrae Radiomics Model | | | |
| --- | --- | --- | --- |
| Selected radiomics feature | Feature class | Filter | LASSO coefficient (β) |
| Large Dependence Low Gray Level Emphasis | GLDM | log.sigma.2.0.mm.3D | -0.527792299 |
| Maximum | First-order | Wavelet (HLL) | -0.427895421 |
| Run Entropy | GLRLM | Logarithm | -0.390672021 |
| Range | First-order | Gradient | -0.370956434 |
| Small Area High Gray Level Emphasis | GLSZM | Logarithm | -0.277695952 |
| Busyness | NGTDM | Log.Sigma.2.0.Mm.3D | -0.187165544 |
| Zone Variance | GLSZM | Log.Sigma.1.0.Mm.3D | -0.139981856 |
| Long Run Low Gray Level Emphasis | GLRLM | Log.Sigma.1.0.Mm.3D | -0.097275185 |
| Long Run Low Gray Level Emphasis | GLRLM | Log.Sigma.2.0.Mm.3D | -0.068798448 |
| Maximum | GLRLM | Wavelet (LLL) | -0.036160636 |
| Median | First-order | Log.Sigma.1.0.Mm.3D | 0.064232295 |
| Short Run High Gray Level Emphasis | First-order | Wavelet (LHL) | 0.151666285 |
| Minimum | GLRLM | Log.Sigma.1.0.Mm.3D | 0.201090538 |
| Root Mean Squared | First-order | Squareroot | 0.44896344 |
| Maximum | First-order | Logarithm | 1.524045047 |

| **Table S3** Selected Radiomics Features and Their Weights for Trabecular Radiomics Model | | | |
| --- | --- | --- | --- |
| Selected radiomics feature | Feature class | Filter | LASSO coefficient (β) |
| Imc1 | GLCM | Wavelet (HLL) | -0.1918093 |
| Kurtosis | First-order | Wavelet (LHL) | -0.0702156 |
| Cluster Prominence | GLCM | Log.Sigma.2.0.Mm.3D | -0.0607821 |
| Zone Variance | GLSZM | Log.Sigma.4.0.Mm.3D | -0.0252812 |
| 90Percentile | First-order | Wavelet (HLL) | 0.00717956 |
| 90Percentile | First-order | Log.Sigma.3.0.Mm.3D | 0.04843617 |
| Interquartile Range | First-order | Gradient | 0.08471728 |
| 90Percentile | First-order | Log.Sigma.2.0.Mm.3D | 0.30455824 |
| Mean Absolute Deviation | First-order | Wavelet (HLL) | 0.31904879 |
| Root Mean Squared | First-order | Square root | 0.32740592 |
| Minimum | First-order | Log.Sigma.5.0.Mm.3D | 0.3831953 |
| Mean | First-order | Log.Sigma.5.0.Mm.3D | 0.50331753 |

**Reference**

1. Milletari F, Navab N, Ahmadi S A. V-net: Fully convolutional neural networks for volumetric medical image segmentation[C]//2016 fourth international conference on 3D vision (3DV). IEEE, 2016: 565-571.
2. Hatamizadeh A, Tang Y, Nath V, et al. Unetr: Transformers for 3d medical image segmentation[C]//Proceedings of the IEEE/CVF Winter Conference on Applications of Computer Vision. 2022: 574- 584.
3. Hatamizadeh A, Nath V, Tang Y, et al. Swin unetr: Swin transformers for semantic segmentation of brain tumors in mri images[C]//International MICCAI Brainlesion Workshop. Springer, Cham, 2022: 272-284.
